# Do We Utilize Our Knowledge of the Skin Protective Effects of Carotenoids Enough?

**DOI:** 10.3390/antiox8080259

**Published:** 2019-07-31

**Authors:** Anamaria Balić, Mislav Mokos

**Affiliations:** 1 Department of Dermatology and Venereology, University Hospital Centre Zagreb, School of Medicine, University of Zagreb, Šalata 4, 10 000 Zagreb, Croatia; 2 School of Medicine, University of Zagreb, Šalata 3, 10 000 Zagreb, Croatia

**Keywords:** antioxidant, skin health, skin aging, skin cancer, photocarcinogenesis, oral photoprotection, nutraceuticals, cosmeceuticals

## Abstract

Due to their potential health-promoting effects, carotenoids have drawn both scientific and public attention in recent years. The primary source of carotenoids in the human skin is diet, mainly fruits, vegetables, and marine product, but they may originate from supplementation and topical application, too. In the skin, they accumulate mostly in the epidermis and act as a protective barrier to various environmental influences. Namely, the skin is exposed to numerous environmental factors, including ultraviolet radiation (UVR), air pollution, and smoking, that cause oxidative stress within the skin with consequent premature (extrinsic) aging. UVR, as the most prominent environmental factor, may cause additional detrimental skin effects, such as sunburn, DNA damage, and skin cancer. Therefore, photoprotection is the first line intervention in the prevention of premature aging and skin cancer. Numerous studies have demonstrated that carotenoids, particularly β-carotene, lycopene, lutein, and astaxanthin, have photoprotective effects, not only through direct light-absorbing properties, but also through their antioxidant effects (scavenging reactive oxygen species), as well as by regulation of UV light-induced gene expression, modulation of stress-dependent signaling, and/or suppression of cellular and tissue responses like inflammation. Interventional studies in humans with carotenoid-rich diet have shown its photoprotective effects on the skin (mostly by decreasing the sensitivity to UVR-induced erythema) and its beneficial effects in prevention and improvement of skin aging (improved skin elasticity and hydration, skin texture, wrinkles, and age spots). Furthermore, carotenoids may be helpful in the prevention and treatment of some photodermatoses, including erythropoietic protoporphyria (EPP), porphyria cutanea tarda (PCT) and polymorphous light eruption (PMLE). Although UVR is recognized as the main etiopathogenetic factor in the development of non-melanoma skin cancer (NMSC) and melanoma, and the photoprotective effects of carotenoids are certain, available studies still could not undoubtedly confirm the protective role of carotenoids in skin photocarcinogenesis.

## 1. Introduction

**Figure 1:**
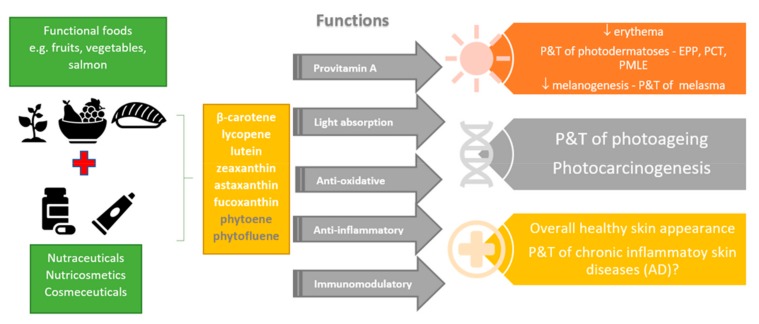
Diverse skin health-promoting effects of food rich in carotenoids or their supplementation through nutraceuticals or cosmeceuticals. P&T, prevention and treatment; EPP, erythropoietic protoporphyria; PCT, porphyria cutanea tarda; PMLE, polymorphous light eruption; AD, atopic dermatitis.

Skin acts as a protective barrier against various environmental influences such as mechanical damage, noxious substances, microorganisms, free radicals, and ultraviolet radiation (UVR). In addition to endogenous factors, external stressors, primarily UVR, result in the alterations of the skin such as inflammation, impaired immune function and epidermal barrier homeostasis, photoaging, and most importantly the formation of various skin diseases and malignancies [1,2]. Due to the diverse proven health-promoting effects, carotenoids are along with some other nutraceuticals widely investigated and put in the focus of interest of many scientific and health-promoting groups [3,4,5,6,7]. We have also noticed a daily increase in the number of analytical techniques for carotenoids determination [8,9]. Due to their vast abundance, especially in plant-derived food, they are an integral part of the human diet. Although carotenoids have never been identified as being essential for humans as long as preformed vitamin A is available through the diet [10,11], the increasing evidence of their important role in the biology and human health continues to stimulate broad interest in the carotenoid field. Consumption of food products and supplements containing carotenoids has increased tremendously due to their health- and skin-favorable or disease-preventive effects, especially in UVR protection and consequently in the prevention of photo-induced dermatoses and skin aging (Figure 1).

## 2. Carotenoids

Carotenoids are known as fat-soluble plant pigments widely distributed in nature that provide diverse colors such as yellow, red, and orange to fruits and vegetables [12]. While they are biosynthesized primarily by plants and algae, as well as by fungi and bacteria, we can find carotenoids throughout the animal kingdom and humans due to selective absorption along the food chain [13]. These lipophilic molecules are based on the chemical structure classified as carotenes and xanthophylls [14]. Both classes have a common C40 polyisoprenoid structure containing a series of centrally located, conjugated double bonds which act as a light-absorbing chromophore. Carotenoids that exist as pure non-polar hydrocarbons are referred to as carotenes (α-carotene, β-carotene, and lycopene); on the contrary, xanthophylls (β-cryptoxanthin, lutein, zeaxanthin, astaxanthin) are more polar carotenoids that contain oxygen as a functional group in its structure either as a hydroxyl or keto group as the end group [15]. The presence of a polar group in the structure affects the polarity and biological function of the compounds [16]. The main sources of around 50 carotenoids in the human diet are fruits and vegetables, followed by green leaves and, to a minor extent, some marine products [14,16,17,18]. The transport of carotenoids from the gut occurs on the uptake with chylomicrons into the lymph, followed by the circulation of lipoprotein particles in the blood, and then transportation to various target tissues with large interorgan differences [19,20]. Absorption of carotenoids in the gut is shown to be mediated by both simple diffusion, which is dependent on the concentration gradient, and facilitated diffusion through cholesterol membrane transporters such as scavenger receptor class B member 1 (SR-B1) and a cluster of differentiation 36 (CD 36) [21,22]. Till now, more than 800 carotenoids have been identified, but only several are found in the human organism, including α-carotene, β-carotene, lutein, and lycopene, as well as the zeaxanthin, and α- and β-cryptoxanthin [23,24,25,26]. Among 800 known carotenoids only 20 of them are studied in sufficient depth; thus, it is an area with huge amount of space in front of scientists. Both α- and β-carotenes, and β-cryptoxanthin are provitamin A carotenoids with different percentage of provitamin A activity [27]. Among provitamins, β-carotene is the most common carotenoid in the diet of mammals and has the highest conversion efficiency to vitamin A with no difference between naturally occurring and chemically synthesized β-carotene. Some of the absorbed β-carotene is cleaved by the enzyme β, β–carotene-15,15′-monooxygenase 1 (BCMO1) into two molecules of all-*trans*-retinal which can be either further reduced reversibly or oxidized. Various fruits and vegetables are rich in carotenoids [28,29,30,31,32,33,34], especially lycopene, such as tomatoes, asparagus, pink grapefruit, guava, and watermelon. Pumpkin, carrots, sweet potatoes, mangos, and papaya are some examples of β-carotene containing food. Oranges, tangerines, nectarines, mango, and papaya are rich in cryptoxanthin. We can find lutein and zeaxanthin in leafy green vegetables, pumpkins, and red peppers. Marine food like microalgae, yeast, salmon, trout, krill, shrimp, crayfish, and crustacea are known as sources of astaxanthin, a strong photoprotectant which has been attributed an enormous potential for protecting the organism against a wide range of diseases due to its strong antioxidant and anti-inflammatory effects [35,36,37,38,39,40]. We also need to mention fucoxanthin, another marine carotenoid with remarkable biological properties that is found in the marine macroalgae—brown seaweeds, and the microalgae—diatoms [41].

The way of food processing affects available carotenoid contents [42,43]. Their bioavailability varies from ~10% in raw materials up to about 50% in commercial and oil-based products [16]. For example, the content of available lycopene is higher in cooked tomatoes and is also increased by the addition of oil, such as olive oil [12,44]. The biological properties of carotenoids are manifold. Besides their natural role as pigments, provitamins, and photosynthetic organisms, they have been demonstrated to possess numerous health-promoting effects as being efficient antioxidants (AOs) – by decreasing reactive oxygen species (ROS) [3,6,45]. However, some carotenoids, especially highly concentrated carotenes, showed pronounced pro-oxidative effects [17,46,47]. It is known that singlet oxygen quenching ability of various carotenoids in organic solvents increases with an increasing number of conjugated double bonds [48]. The biologically important shorter chain C40 carotenoids have quenching rate constants half those of longer chain carotenoids such as β-carotene; e.g., in the case of lutein with ten double bonds the quenching rate is lower than in those with 11 double bonds such as β-carotene or zeaxanthin [49].

## 3. Carotenoids and the Skin

The amount of carotenoids in the skin depends on dietary intake or supplementation, and their bioavailability from various food [50]. After absorption in the gut and transportation into the skin, carotenoids accumulate mainly into the epidermis. It is thought that they are transported into the epidermis by the same prior mentioned cholesterol transporters—SR-B1 found in the basal layers because the epidermis is an active site of cholesterol accumulation crucial for the epidermal barrier function [51]. Due to the accumulation of carotenoids in the epidermis, in the cases of excessive carotenoid intake, mostly β-carotene, carotenoderma occurs as yellowish discoloration of the skin being most obvious on the palms and soles [52,53]. Besides the oral route of carotenoid administration, topical application is just as important, being especially of great interest for cosmetic companies [54,55,56,57,58]. Topical application of antioxidant (AO) substances such as carotenoids is closely related to skin protection from environmental factors and anti-aging [59,60]. To determine the total dermal carotenoid level in comparison with their respective plasma levels, Scarmo et al. [61] performed skin biopsies of healthy individuals and collected their blood samples for correlation of individual and total carotenoid content using high-performance liquid chromatography (HPLC). Later on, this group of authors in their studies used less invasive methods such as a resonance Raman spectroscopy (RRS) to assess carotenoid status in human tissues [62]. Based on their findings, it is suggested that β-carotene and lycopene are present in greater abundance in human skin, in comparison to zeaxanthin and lutein, possibly indicating a specific role of carotenes in human skin photoprotection. Interestingly, levels of carotenoids in skin differ within the skin layers and the body locations with the highest levels in the skin of palms, forehead, and dorsal skin [63]. The lifestyles of individuals also reflect carotenoid levels in the skin; precisely, stressors like UVR, illness, smoking, and alcohol consumption lower their concentrations [64]. As more and more topical products and oral supplements with antioxidative effects are occurring on the market, Darvin et al. wanted to examine in vivo whether the topical, systemic or combined application of AOs/carotenoids is effective in increasing their concentration in the skin by using non-invasive RRS [65]. In this study, carotenoids were applied systemically (carotenoid tablets) at physiological concentrations like those contained in a healthy diet, and topically (cream) in the concentrations corresponding to those physiologically present in healthy skin. Results of this study showed a statistically significant increase of AOs levels in human skin with all forms of treatments—topical, systemic, and combined. A combination of topical and systemic AOs induced the highest accumulation in the skin, which suggested that combined treatment might be an optimal form of protection of the human skin. Carotenoid levels after the end of treatment were preserved for about two weeks following the topical application, and up to five weeks after systemic administration. These results are explained by the fact that topically applied AOs are stored in *stratum corneum* for a short time only due to their rapid depletion by skin desquamation, textile contact, washing, and environmental stress. On the other hand, the systemically applied carotenoids are stored in body fat tissue and slowly release onto the skin surface with sweat and sebum. Based on these findings, it could be concluded that the combined topical and systemic application of carotenoids/AOs represents an optimal form of skin protection, but we need to be aware of the importance of choosing appropriate non-lipid formulation of topical product which does not saturate reservoir of *stratum corneum*, thus allowing the systemically applied carotenoids to penetrate back into the skin.

## 4. Carotenoids in Skin Photoprotection

People are constantly exposed to ultraviolet (UV) light, some less, some more, depending on the place of living, activities, what they do for a living, hobbies, culture, but also their knowledge of the importance of sun protection and its implementation. It has been estimated that the exposure to solar UV light is ~10% of the total available annual UVR for outdoor workers and ~3% for indoor-working adults [66]. Besides its few beneficial health effects, including vitamin D3 synthesis, improvement of mood through production of endorphins, efficacy in the treatment of various skin diseases, such as psoriasis, vitiligo, and atopic dermatitis; UVR causes many detrimental skin effects—sunburn, ocular damage, photoaging, immune suppression, DNA damage and skin cancer [66,67,68,69,70]. Sun exposure results in photoaging—solar elastosis, skin roughness, furrows, and wrinkles by the mechanisms of mitochondrial deletion and remodeling of the extracellular matrix (ECM) mediated through matrix metalloproteinases (MMP) which cause the damage of collagen and elastin fibers [71]. Most importantly, UVR plays the main role in photo-induced carcinogenesis, melanoma, and non-melanoma skin cancer occurrence [69]. Most of the harmful effects of UVR are mainly mediated by oxidative stress which alters signal transduction pathways such as the nuclear factor-kappa beta (NF-κB)/p65mitogen-activated protein kinase (MAPK), the janus kinase (JAK), signal transduction and activation of transcription (STAT), and the nuclear factor erythroid 2-related factor 2 (Nrf2), causing the damage to biomolecules and affecting the integrity of skin cells leading to skin damage [71]. UVR also induces pro-inflammatory genes and causes immunosuppression by reducing the number and activity of the epidermal Langerhans cells [72].

Many skin diseases form as a result of pathological processes induced by photo-oxidative damage. UVA radiation, which contributes to up to 95% of total UV radiation, does not interact with DNA; however, it is considered the most important source of oxidative stress in human skin. As UVA radiation penetrates the deeper dermis, it plays a significant role in photoaging [73]. On the other hand, UVB radiation is mainly absorbed by keratinocytes in the epidermis and interacts directly with DNA, causing mutations and skin cancer [69,71]. UVB is the leading cause of sunburn, erythema resulting from an inflammatory response to the photodamage of the skin [74]. In the last decade it has been discovered that visible light (400–700 nm) causes solar erythema, thermal damage, induces the melanogenesis in human skin [75] but also contributes to signs of premature photoaging by inducing production of ROS, proinflammatory cytokines, and MMP-1 expression [76]. Additionally, visible light exposure is related to the pathogenesis of some photodermatoses [77].

Photoprotection, either mechanical or pharmacological, is the first line in the prevention of photoaging and skin cancer. Pharmacological photoprotection can be topical or systemic. The main principle of photoprotection is the direct absorption of UV light using suitable compounds. There is an increasing interest in the area of skin protection from UV and visible light by additional endogenous protection by dietary micronutrients with AO properties such as carotenoids, vitamin C and E, and polyphenols [78,79,80,81]. Skin photoprotection by nutritional means [54,63,82,83,84,85] or topically-applied phytochemicals has been examined by various authors [86,87]. Human interventional studies have documented photoprotective effects of many carotenoids, particularly β-carotene, but also lycopene, lutein, and astaxanthin, provided through topical application, or orally, either by a carotenoid-rich diet or by supplementation, but rather long treatment periods with a minimum of 10 weeks were required [37,47,59,79,88,89]. Most of the carotenoids exhibit absorbance maximum at wavelengths in the range of visible light. However, noncolored carotenoids phytoene and phytofluene have high UV absorption maxima that cover both UVB and UVA range. Lutein and zeaxanthin may protect the skin from blue light, which makes them useful in the prevention or treatment of melasma and in ocular protection. [90,91,92,93]. Besides direct light-absorbing properties, carotenoids and some other micronutrients provide endogenous photoprotection and contribute to the prevention of UV damage in humans mostly by their well-known AO effects—scavenging ROS, including excited singlet oxygen and triplet state molecules which would lead to photoinactivation of AO enzymes, lipid peroxidation, and DNA damage induction [63,71,78,94]. Additionally, they interfere with UV light-induced gene expression by multiple pathways, modulate stress-dependent signaling, and/or suppress cellular and tissue responses like inflammation [63,95,96,97]. The idea of endogenous photoprotection implies that the active compound is available in sufficient amounts at the target site [63]. That is why structural features of carotenoids are important because they influence pharmacokinetic parameters like absorption, distribution, and metabolism and affect the level of the active compound in the skin [11,98,99].

Interventional studies in humans with carotenoid-rich diet have shown its photoprotective effects on the skin, mostly by decreasing the sensitivity to UV radiation-induced erythema. See Table 1.

### 4.1. Lycopene

Lycopene is considered the most efficient dietary carotenoid when it comes to quenching singlet oxygen in organic solvents [145]. Its quenching efficacy is, in this case, greater than all C40 carotenoids and twice greater than the one of β-carotene. On the contrary, others state that lycopene is only slightly more efficient than β-carotene and that in more biomimetic environments, such as micelles and liposomes, lycopene, and β-carotene have rather similar quenching abilities [49]. There are also human cell protection studies of lycopene on oxidant-induced damage, demonstrating its beneficial AO effects and strong protection role in comparison with other carotenoids [146,147,148].

Stahl et al. [149] conducted an interventional study to investigate whether intervention with a natural dietary source rich in lycopene protects against UV-induced erythema in humans. They found that ingestion of tomato paste (40 g per day, equivalent to 16 mg lycopene per day) with 10 g of olive oil over ten weeks led to 40% reduction of skin erythema induced by exposure to solar-simulating UVR. No significant protection was found after four weeks of dietary intervention, but after ten weeks, erythema was significantly lower than in the control group receiving olive oil only. Rizwan et al. [87] previously conducted a similar study where they also examined whether ingestion of 55 mg tomato paste in olive oil daily over 12 weeks can protect human skin against UVR-induced effects—erythema, changes in ECM, and mitochondrial DNA (mtDNA) damage. UVR-induced erythema was assessed visually as the minimal erythemal dose (MED) but also quantitatively with an instrument pre-and-post nutrition rich in lycopene. To demonstrate UVR-induced ECM changes, and mtDNA damage, partially mediated by oxidative stress, they performed skin biopsies from unexposed and UVR-exposed skin before and after the nutritional intervention. Skin samples were further analyzed immunohistochemically for MMP-1, fibrillin-1, and procollagen I, and by quantitative polymerase chain reaction (PCR) for mtDNA bp deletion. Based on these and previously-mentioned study results, it is reasonable to conclude that the consumption of food rich in lycopene protects against acute and potentially longer-term aspects of photodamage.

### 4.2. Lutein

Lutein is also an efficient singlet oxygen quencher, though it is less efficient than lycopene and β-carotene [145,150]. Besides the well-examined photoprotective role of lycopene, Grether-Beck et al. [79] wanted to investigate the skin protective effects of lutein against UVR on a molecular basis. Their double-blind, randomized, controlled study added a fact that besides lycopene-rich tomato nutrient complex (TNC), lutein protects from UVA/B- and UVA1-induced gene expression in human skin. Assuming the role of heme-oxygenase 1, intercellular adhesion molecule 1 (ICAM-1) and MMP-1 mRNA as indicators of oxidative stress, photodermatoses, and photoaging, these study results indicate that TNC and lutein could protect against UVR-induced skin damage.

### 4.3. β-Carotene

Most studies that describe the role of carotenoids in photoprotection investigated the photoprotective role of β-carotene and its effectiveness in the prevention of UV-induced erythema formation, being especially useful in the treatment and prevention of some photodermatoses, namely EPP and PMLE [83,88,89,100,113,114,151,152]. Systemic photoprotective effects of this provitamin depend both on the dose and the duration of treatment. In most of the interventional studies with carotenoids, photoprotection was observed only after a minimum of 10 weeks of dietary intake or supplementation, with doses >12 mg/day [89,101,153]. A sufficiently long period of treatment is needed to provide optimal photoprotection of the skin. The photoprotective role of β-carotene is fortified also in vitro by the findings that its supplementation significantly reduces the rate of mtDNA mutation in human dermal fibroblasts after UVR [117]. However, the need for caution with isolated β-carotene supplementation was pointed up after human interventional trials that had demonstrated potentially harmful effects of high dosages of this carotenoid and raised a discussion on suitable dose amounts for photoprotection. In two long-term interventional trials in individuals at high risk for cancer (cigarette smokers and asbestos workers) who received β-carotene for several years at doses of 20 and 30 mg/day, there was an ~20% increase in the incidence of lung cancer [6,154]. The authors concluded that the effects of higher doses of β-carotene in cancer pathogenesis could partly be explained by the formation of eccentric cleavage products of β-carotene, which can interfere with the retinoic acid receptor-mediated signaling pathway [27]. Despite these interventional trials results, dermatologists are still encouraged to recommend β-carotene supplements for photoprotection, especially in patients with EPP, photosensitive diseases, and to reduce the phototoxic damage caused by some drugs [155].

### 4.4. Astaxanthin

In recent years, much attention has been put on the health and skin benefits of astaxanthin [35]. Astaxanthin, a marine pigment, is mostly produced by the microalga *Haematococcus pluvialis* to protect its cells from sun radiation, UV-light, and oxidation [156]. Camera et al. [37] conducted a study in which they examined the modulation of UVA-related injury by astaxanthin, canthaxanthin, and β-carotene for systemic photoprotection in human dermal fibroblast. In this study, astaxanthin showed significant photoprotective effect and counteracted UVA-induced alterations to a greater extent. The uptake of astaxanthin by dermal fibroblasts was higher than that of other two carotenoids, which led to the assumption that the antioxidative photoprotective effect of astaxanthin was stronger than of the other substances. Other in vitro studies fortified the photoprotective role of astaxanthin by showing that it could interfere with UVA-induced MMP-1 and skin fibroblast elastase/neutral endopeptidase expression [135,157].

### 4.5. Fucoxanthin

Similar to astaxanthin, orange-colored pigment accumulated by marine plants, which has AO and provitamin A effects, fucoxanthin, shows a protective effect against UVB-induced skin damage by decreasing intracellular ROS [40,158]. Matsui et al. suggest that this skin sun-protective effect may be due to the restoration of filaggrin and promotion of skin barrier formation, unrelated to AO action [159]. As inherited or acquired filaggrin deficiency substantially contributes to the pathogenesis of atopic dermatitis, fucoxanthin might be useful in its and similar conditions treatment. Besides the sun-protective effect, fucoxanthin exhibits anti-pigmentary activity in UVB-induced melanogenesis either by oral or topical route of application presumably by the suppression of melanogenic stimulant receptors and prostaglandin E2 synthesis [160].

## 5. Carotenoids and Photocarcinogenesis

Based on the above statements of the skin photoprotective role of carotenoids, a question about their relationship with skin cancer incidence pops up. Regardless of their proved role as agents that prevent skin cancer in vitro and in animal studies; human interventional or epidemiological studies regarding the effect of carotenoids on the incidence of UV-induced skin cancer are lacking (see Table 1). However, the study conducted by Heinen et al. [142] among Australian population showed that high dietary intake of lutein and zeaxanthin was related with a decreased incidence of SCC in persons who had a history of skin cancer.

## 6. Carotenoids and Skin Aging

A healthy diet based on large amounts of fruits and vegetables is known to be beneficial in the prevention of skin aging, especially photoaging, as it increases the concentration of AOs in the blood and the skin substantially [65,161,162,163,164].

### 6.1. Lycopene

Meinke et al. [164] measured the blood and skin levels of the carotenoids in individuals after oral administration of natural kale extract, or placebo oil, for four weeks. Carotenoid bioaccessibility was evaluated using RRS on the forehead skin and the palm. For the analysis of the blood serum, the standard HPLC method was employed. In this study, carotenoids’ bioaccessibility increased significantly in both skin and serum, but increases in the skin were delayed when compared with serum levels and depended on the dermal area as well as on the type of carotenoid. Lycopene bioaccessibility increased more in the skin compared to the blood, which indicates that the natural kale extract stabilizes the AO network in the skin. Carotenoids’ levels decreased significantly faster in the blood than in the skin after the end of supplementation, which may indicate a peripheral buffer function of the skin for carotenoids. It is shown that individuals with a higher concentration of lycopene in the skin have a significantly smaller amount of wrinkles and furrows than individuals with lower concentrations which fortifies the protective role of lycopene when it comes to pro-oxidative damage [101,123]. It has also been demonstrated that the skin roughness is reduced after systemic application of carotenoids [44,165].

### 6.2. Lutein and Zeaxanthin

Some studies have examined the efficacy of lutein and zeaxanthin, found naturally in the skin, upon several skin physiology parameters. A group of Italian authors [54] designed randomized, double-blind, placebo-controlled, 12-week clinical multicenter study to evaluate the effect of lutein and zeaxanthin administered both orally and topically upon human skin of forty healthy middle-aged women that exhibited signs of premature skin aging. The study results showed that the provision of the previous two carotenoids ensures multiple benefits to the skin. Besides the prevention of UVR-inducible damage, these xanthophylls also improved skin features—skin hydration, elasticity, and increased skin surface lipids. These beneficial skin effects were achieved regardless of whether both xanthophylls were administered topically or orally, which demonstrates that the simultaneous administration of these carotenoids by both routes could result in greater skin health. Similar findings were observed in a study observing the skin effects of only zeaxanthin both topically and/or orally [144]. Meinke et al. recently fortified their prior finding of the beneficial role of carotenoids in the prevention of skin aging by showing that a natural carotenoid-rich extract could prevent the aging-related collagen I degradation in the dermis and improve the ECM [166]. In their study, 29 healthy middle-aged female volunteers received a supplement in the form of a carotenoid-rich natural curly kale extract containing 1650 µg of carotenoids in total (three capsules of 550 µg), once a day for up to 10 months. Their skin was examined in vivo using noninvasive RS-based scanners and two-photon tomography for determination of skin carotenoids before, after five months, and after ten months of daily supplementation. The results showed a significant increase in skin carotenoids and the collagen I/elastin aging index of the dermis proportional to the duration of carotenoid supplementation.

### 6.3. β-Carotene

Compared to a vast number of experimental studies investigating the effectiveness of β-carotene in the prevention of UV-induced erythema and skin photodamage, there are only a few clinical studies investigating its role in photoaging. One of them is a Korean study in which was conducted with the aim to determine the effects of 90 days supplementation with two different doses of dietary β-carotene (30 and 90 mg/day) on UV-induced DNA damage in human skin in vivo, procollagen type I, MMP-1, and fibrillin-1 gene expression, and skin elasticity and wrinkle formation [167]. Their findings led to a conclusion that low-dose β-carotene supplementation prevents and repairs photoaging, which reflects as improvement in facial wrinkles and elasticity. However, other clinical studies have failed to convincingly demonstrate its beneficial effects [168].

### 6.4. Fucoxanthin

Fucoxanthin may be an active ingredient of cosmeceuticals and nutraceuticals used in the protection of the skin from photoaging [169]. Its beneficial role in skin aging protection is based on the findings of Urikura et al. [170] which demonstrate that fucoxanthin significantly suppressed UVB-induced wrinkle formation, epidermal hypertrophy, MMP-13, vascular endothelial growth factor (VEGF) and the increase of other reactive substances in the UVB-irradiated animal model of hairless mice.

### 6.5. Astaxanthin

As we mentioned in the previous section, astaxanthin is among the carotenoids considered to be a potent skin protective nutrient due to its natural capacity to protect cells from irradiation and oxidation, proven to prevent or minimize the signs of UVB-induced skin damage but also UVA-induced photoaging such as skin wrinkling or sagging by topical or oral administration routes [35,37,133,157]. Several human studies, which were later confirmed in animal studies [135,171], demonstrated that astaxanthin, in addition to improvement of the appearance of wrinkles, also improved skin elasticity, moisture, age spots, and skin texture [172,173]. In vitro studies have demonstrated that astaxanthin improves the function of mitochondria and has protective effects on human skin fibroblasts by exhibiting other biological functions rather than AO, including effects on gap junctional communication important for homeostasis, growth control, and development of cells [174]. In that way, it can protect skin cells from ROS and preserve the collagen, which results in the smooth and youthful appearance of the skin. Based on the results of numerous studies, it is reasonable to conclude that astaxanthin supplementation has promising functional improvements to the skin and that it can help reduce the skin aging process (see Table 1).

## 7. Future Perspectives

Available data on the protective effects of carotenoids on human skin may encourage their implementation in the field of dermatology as nutraceuticals, cosmeceuticals, and photoprotectants. Due to their antioxidant, anti-inflammatory, and immunomodulatory effects, the optimal supply of these micronutrients increases dermal defense against UVR, maintains longer-term protection, alleviates certain photodermatoses, and contributes to better skin health and appearance. Hopefully, besides their beneficial role in reducing late effects of UVR such as photoaging, carotenoids may be helpful in the treatment of inflammatory skin diseases like atopic dermatitis and psoriasis. If so, their use could be beneficial at a population level or at least in those perceived to be at high risks, such as outdoor workers, immunosuppressed patients, patients with various photodermatoses other than PMLE, EPP, and porphyria cutanea tarda, but also ones requiring repeated courses of phototherapy. In practice, advice on achieving a proper intake of protective carotenoids should probably emphasize natural food sources rather than supplements. As the diet rich in fruits and vegetables is already being encouraged in many countries due to its various protective health effect, it should be a public health message in each state. As nanotechnology is a promising field of research for the development of nutrient delivery systems, future interventions in the area of carotenoid micronutrition and cosmetics would be of benefit for dermatology patients. Efforts are also needed to enhance the knowledge of already known and still undiscovered health-promoting effects of various carotenoids along with other antioxidants, but also in the development of new topical and systemic photoprotective drugs. We hope that the application and advancements in nutritional and topical skin protection, and in new technologies will enable us to utilize more knowledge of the skin protective effects of carotenoids when approaching our patients.

## Figures and Tables

**Table 1:** Carotenoids in skin health, repair, and disease—summary.

Carotenoid	Food Source	Photoprotective Effects	Role in Photo-Induced Carcinogenesis Prevention	Role in Photoaging Prevention	Additional Benefits
β-carotene	Pumpkin, carrots, sweet potatoes, mangos, papaya, bilberry [28,29,30,33]	Prevention of UV-induced erythema [88,100,101], ↑^1^ MED^2^ [102,103], ↓^3^ of the rate of mitochondrial mutation in human dermal fibroblasts after UV irradiation [96]	Delayed tumor appearance and reduced tumor growth rates [104], inhibition of photocarcinogenic enhancement by benzopyrene [105], in vitro induction of apoptosis of melanoma cells by activation of caspase-3,-8, and -9 [106] or by additional regulation of Bcl-2, p53 [107] In vivo no influence, positive or negative, on the incidence of malignant skin neoplasms, including melanoma. and NMSC^4^ [108,109,110]	O2 quenching,↓MMP^5^-1, -3, and MMP-10 [96,111],↓MMP-9 partly, by inhibiting Chol-OOHs^6^ formation [112]	Combination of β-carotene, lycopene and *Lactobacillus johnsonii* inhibits PMLE^7^ [113], protective role in the treatment of EPP^8^ and PCT^9^ by membrane protection against protoporphyrin IX and uroporphyrin I [114,115,116]
Lycopene	Tomatoes, asparagus, pink grapefruit, guava, watermelon, peaches, papaya [28,29,33]	Protection against UV-induced erythema [95,117,118,119] ↓ HO-1^10^, ↓ ICAM-1^11^ [79]	Inhibits mtDNA deletion [87], inhibits skin tumor formation [120], induction of apoptosis [121], chemoprevention properties in photocarcinogenesis remain contradictory [122]	↓ MMP [79], ↓MMP-1 and ↓ reduction in fibrillin-1 [87],↓ amount of furrows and wrinkles [101,123]	PMLE prevention [113], protective role in EPP [115]
Astaxanthin	Microalgae, yeast, salmon, trout, krill, shrimp, crayfish and crustacea [28,29,35,39]	Protection against UV-induced erythema, ↑MED, activation of Nrf213/HO-1 AO pathway [35,37,38,124]	Inhibition of skin cancer and tyosinase in rat model [125]; apoptosis [126]; AO effect, effect on gap junctional communication important for homeostasis, growth control, and development of cells [127,128,129], may enhance immune responses and potentially exert antitumor activity [130]	↓ wrinkle parameters [131,132], ↑ elasticity, improved skin texture, and ↓ TEWL12 [38,131,133,134], ↓ size of age spots [131], ↑ procollagen type I, ↓MMP-1, -3, -12, also MMP-13 [126,133,135], ↓ malondialdehyde; ↓ residual skin surface components [136,137], ↓ IL14-1α [132], ↓ MIF15, IL-1β, TNF-α16, preserves trans-UCA17 levels [126], ↓ mast cells [135]	Anti-inflammatory properties - ↓ iNOS18, COX-2, and inhibition of NFκB signaling [138]; ↓ TNF-α, IL-1β, IL-6— possible implication for the treatment of inflammatory diseases such as atopic dermatitis [138] and psoriasis Accelerates wound healing—↓iNOS, ↑wound healing biological markers including Col1A121 and bFGF22 [139]
Lutein/ Zeaxanthin	Leafy green vegetables, peas, broccoli, pumpkins, corn, red peppers, egg yolk, bilberry [28,29,30,34]	↓ skin edema and erythema after UVR [140], ↓ masts cells number [141], ↓ melanogenesis [93], blocking of eye damage induced by blue light [90,91]	↑ tumor-free survival time, ↓ tumor volume and multiplicity [141], ↓ PCNA^23^ and BrdU + epidermal cells [140], reduced incidence of SCC^24^ in persons who had a history of skin cancer at baseline [142]	↓overexpression of HO-1, ICAM-1, MMP-1 Genes [79], ↓ MMP-1 and MMP-7, ↑ TIMP-2 [141,143], ↑ surface lipids, skin hydration, and skin elasticity [54,144]	Prevention of melasma, skin-lightening effects [93]

^1^ increase, ^2^ minimal erythema dose, ^3^ decrease, ^4^ non-melanoma skin cancer, ^5^ matrix metalloproteinase, ^6^ cholesterol hydroperoxides, ^7^ polymorphous light eruption, ^8^ erythropoietic protoporphyria, ^9^ porphyria cutanea tarda, ^10^ heme oxygenase-1, ^11^ intercellular adhesion molecule, ^12^ transepidermal water loss, ^13^ nuclear factor erythroid 2-related factor 2, ^14^ interleukin, ^15^ macrophage migration inhibitory factor, ^16^ tumour necrosis factor-alpha, ^17^ trans-urocanic acid, ^18^ inducible nitric oxide, ^19^ cyclooxygenase-2, ^20^ nuclear factor-kappa beta, ^21^ collagen type I alpha 1 chain, ^22^ fibroblast growth factor, ^23^ proliferating cell nuclear antigen, ^24^ squamous cell carcinoma, ^25^ tissue inhibitor of metalloproteinase-2.
